# A Multimetal Approach for the Reticulation of Iridium into Metal–Organic Framework Building Units

**DOI:** 10.1021/jacs.4c08638

**Published:** 2024-09-04

**Authors:** Raluca Loredana Vasile, M. Carmen Borrallo-Aniceto, Fátima Esteban-Betegón, Alina A. Skorynina, Miguel Gomez-Mendoza, Victor A. de la Peña O’Shea, Enrique Gutiérrez Puebla, Marta Iglesias, M. Ángeles Monge, Felipe Gándara

**Affiliations:** † Materials Science Institute of Madrid − Spanish National Research Council (ICMM-CSIC), 28049 Madrid, Spain; ‡ CELLS-ALBA Synchrotron Radiation Facility, 08290 Barcelona, Spain; § Photoactivated Processes Unit, IMDEA Energy Institute, Ramón de la Sagra 3, 28935 Móstoles, Spain

## Abstract

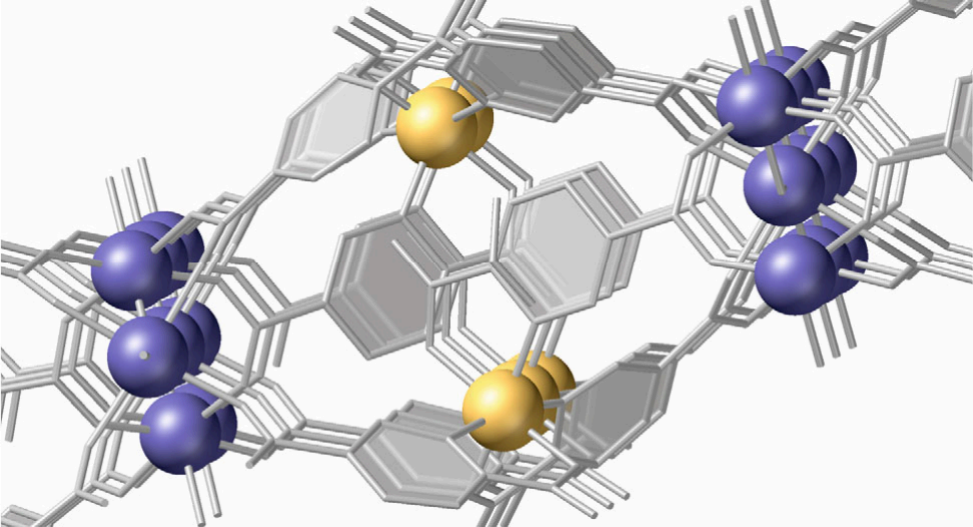

Noble metal elements are ubiquitous in our everyday life, from medical applications to electronic devices and synthetic chemistry. Iridium is one of the least abundant elements, and despite its scarcity, it remains essential for efficient and active catalytic processes. Consequently, the development of heterogeneous catalysts with the presence of active iridium sites is of enormous interest as it leads to the improvement of their recyclability and reusability. Here, we demonstrate a strategy to incorporate iridium atoms into metal–organic frameworks (MOFs), as part of their secondary building units (SBUs), resulting in robust and reusable materials with heterogeneous photocatalytic activity.

## Introduction

Iridium is a noble metal element with very low earth abundance, widely used in a breadth of applications, ranging from solar cells^1^ to organic electronic devices^2^ and biological applications^3−5^ such as anticancer agents^6^ or biological probes.^7^ Iridium-based materials have found great relevance in the catalysis field due to their unique properties and unparalleled activity. Thus, iridium compounds are key catalysts for important chemical transformations, such as water splitting for which IrO_2_ is a stable catalyst, as it can withstand harsh acidic conditions.^8−11^ In addition, iridium molecular catalysts, such as pincer complexes^12^ or *N*-heterocyclic carbenes complexes,^13^ are generally used in the homogeneous phase.^14^ Although it is possible to incorporate molecular iridium compounds onto solid supports^15−17^ and use them under heterogeneous conditions, their use mostly remains restricted to the homogeneous catalysis field. However, the incorporation of iridium atoms with a molecularly tunable environment into the structure of porous solids can provide great advantages for their use as heterogeneous catalysts, among the most important ones being the possibility of recovering and reusing them, thus capitalizing on the costs associated with catalyst synthesis but also being in chemically tunable frameworks with adjustable pore environments, such as those offered by metal–organic frameworks (MOFs). MOFs are a class of reticular compounds that consist of a combination of metal cations or clusters, termed inorganic secondary building units (SBUs), connected by organic linkers, generating extended structures with potential porosity. Over the past two decades, almost every metal element in the periodic table has been used to create MOFs, from alkali metals^18^ to uranium,^19^ with only a few exceptions, including iridium as one of the elements that has not been incorporated yet into the SBU of a MOF.^20^ Although there are examples of the use of iridium in MOFs for important catalytic applications,^21−36^ all of them reported iridium atoms being either incorporated as part of a metalloligands or inserted postsynthetically and coordinated to anchoring groups of the linkers. The direct use of iridium in MOF synthesis remains elusive, likely due to the distinctive coordination chemistry properties of iridium cations, especially concerning the use of carboxylic acid linkers, which remain the most widely used in MOF synthesis due to their high designability in forming inorganic SBUs. Indeed, the use of inorganic SBUs has been crucial in the development of the MOF field, providing the fundamental principles for the rational design of these materials, including reticular synthesis, postsynthetic modifications, or multimetal complexity.^37^ These critical design principles, however, remain untapped with respect to iridium. Considering this, we hypothesized that a multimetal synthetic approach involving the combination of iridium with a second metal element could facilitate the formation of an extended framework featuring iridium-based SBUs. To maintain control over the location and environment of both metal elements, our strategy involved selecting an organic linker that bears two different coordination modes, directing the insertion of each of the two metal elements into specific coordination environments and therefore producing distinct SBUs. As a result of this synthetic strategy, herein we report the preparation and crystal structure of two new MOFs obtained through the combination of iridium with indium or scandium as metal elements. Iridium atoms are oxidized during the synthesis, and for the first time, iridium(IV) cations are thus reticulated into an extended framework by forming inorganic SBUs. The resulting materials are highly robust and stable in both water and air and under acidic and basic conditions. The catalytic activity endowed by the presence of the iridium centers is demonstrated through the photooxidation of sulfides, selectively and quantitatively yielding the corresponding sulfoxides under heterogeneous conditions without a loss of activity in up to six consecutive cycles.

## Results and Discussion

**Scheme 1:**
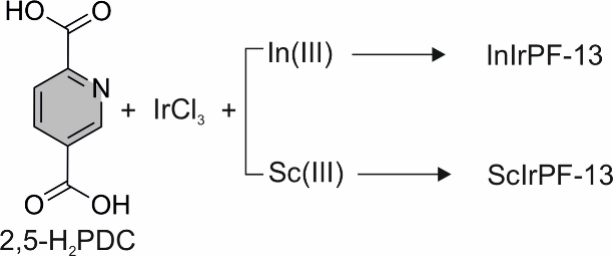
Hydrothermal Reaction of 2,5-H_2_PDC with IrCl_3_ and In(III) or Sc(III) Salts Results in the Obtaining of Novel Iridium MOFs

To implement our synthetic strategy, we settled on the *N*,*O*-donor 2,5-pyridinedicarboxylic acid (2,5-H_2_PDC; Scheme 1), considering that iridium complexes with related pyridinecarboxylic moieties, in which the iridium atoms coordinate to both the pyridinic nitrogen and the carboxylic oxygen atoms are known in the literature.^38−40^ In the present case, this would leave the carboxylate groups at position 5 of the ring available to coordinate to the second metal atom, thus facilitating the formation of a framework. As for the additional metal, we first selected indium, which is a low-toxicity metal that displays variable coordination capabilities, thereby resulting in a rich structural diversity,^41^ and a marked Lewis acid character that makes it widely used in catalysis.^42−46^ Moreover, indium-based MOFs are usually highly stable in air or in a humid environment. This stability can be explained by the electron-rich hypervalent bonding pattern in six-coordinate systems, which has a direct impact on the general features of the materials as they are generally more thermodynamically stable and more reactive from a kinetics point of view.^47^ In addition to indium, we also tested the applicability of our approach with other trivalent cations. In particular, we chose scandium, which is the smallest and least basic rare-earth element, with a strong oxyphilic character for scandium(III), as it tends to coordinate to hard donor atoms like O atoms from carboxylate groups. Similarly to indium, it usually affords highly robust and stable frameworks,^48^ and moreover, there are examples of isostructural MOFs reported with indium or scandium.^42,49^

**Figure 1:**
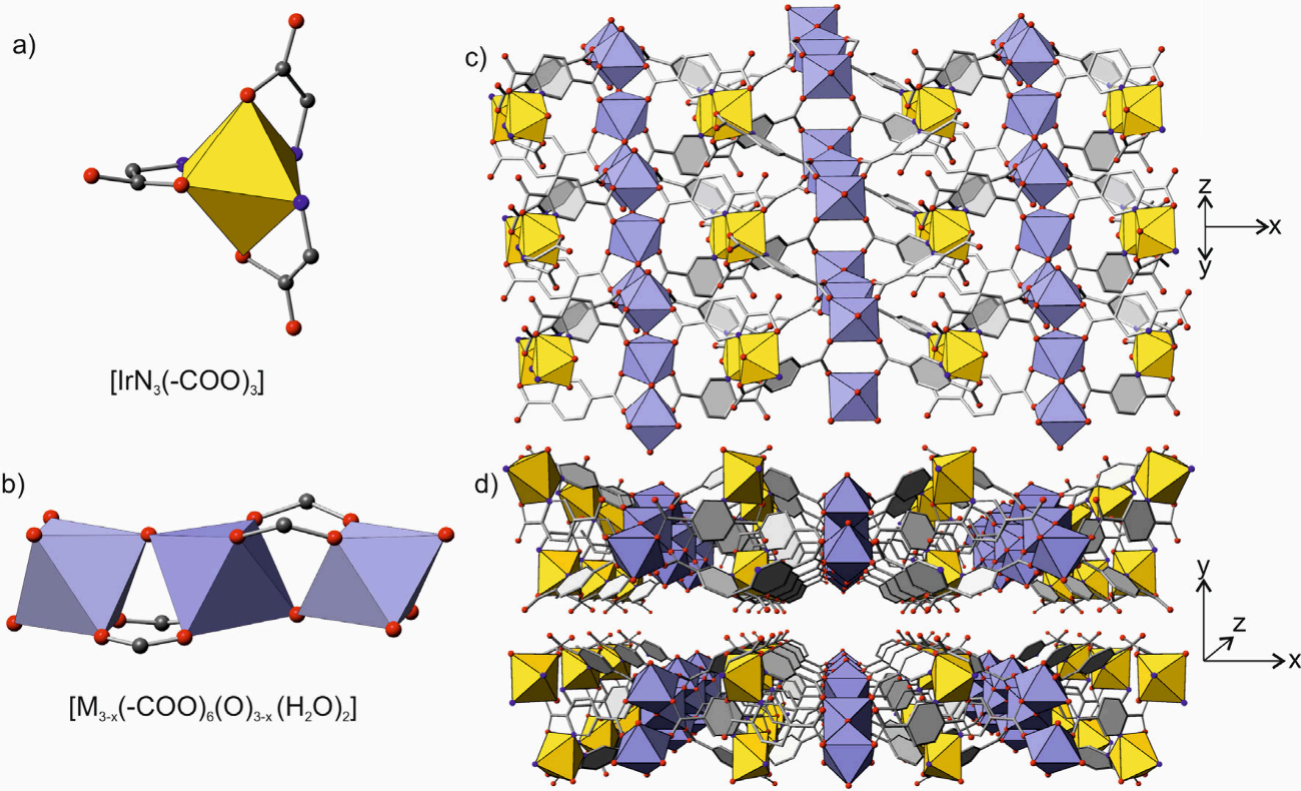
(a) Representation of the coordination environment of the iridium SBU. (b) SBU formed by scandium or indium atoms. The three crystallographic positions are shown in the figure, but only the central one is fully occupied, with the other two sites being disorderly vacant. (c) Top view of one of the layers, formed by the connection between the two types of SBUs. (d) View along the *c* axis, showing the two-dimensional structure, and the oval shaped channels formed in the interlayer space. Occluded water molecules have been omitted for clarity. Yellow and violet polyhedra represent iridium and indium or scandium atoms, respectively. Oxygen and nitrogen atoms are red and blue spheres, and carbon atoms are shown as gray sticks.

Therefore, we found that the hydrothermal reaction of iridium chloride, indium nitrate, and 2,5-H_2_PDC at 150 °C resulted in the formation of a crystalline product, denoted InIrPF-13, while reaction with scandium acetate at 170 °C results in the isostructural material (full synthetic details can be found in the Supporting Information, SI). Single-crystal X-ray diffraction (SCXRD) analysis confirmed the formation of an extended framework through the coordination of both indium or scandium and iridium atoms to the 2,5-PDC linkers. The new compounds, InIrPF-13, and ScIrPF-13 crystallize in the orthorhombic space group *Cmc2*_*1*_ with cell parameters *a* = 25.6795(12) Å, *b* = 23.9298(11) Å, *c* = 9.8202(5) Å, and cell volume 6034.6(5) Å^3^, in the case of InIrPF-13 (full crystallographic parameters and refinement data are displayed in Tables S1, S2). The iridium atoms form an inorganic SBU by bonding via N- and O-heterochelation to three linker molecules in an octahedral geometry (Figure 1a). On the other hand, the scandium or indium atoms form another type of SBU, with μ-O vertex-sharing octahedra (Figure 1b). There are three crystallographically independent indium or scandium positions in the structure. However, careful analysis of the X-ray diffraction data indicates that only one of them is fully occupied, while two other sites are present with only partial occupancies. In the particular case of indium, crystal refinement of the occupancies resulted in a significant improvement of the residual values, with occupancy values of 74 and 48% for the two metal sites. This occupational disorder of the metal atoms is also accompanied by the positional disorder of the carboxylic oxygen atoms involved in their coordination. To corroborate the metal ratios, scanning electron microscopy-energy dispersive X-ray spectroscopy (SEM-EDX, Figures S1–S4) analysis was conducted on several samples. Thus, a total of 35 analyses were completed on 10 different samples of InIrPF-13, finding In/Ir ratios in the 1.5–0.8 range, with an average of 1.2, this value being the most commonly observed. This variability in the indium content demonstrates the intrinsically defective nature of this SBU in the MOF. In the case of ScIrPF-13, the corresponding SEM-EDX study involving a total of 14 analyses showed a similar average Sc/Ir ratio of 1.2, indicating that metal-site vacancies are also present in this inherently disordered SBU. Metal ratios in the bulk samples were determined with the use of total reflection X-ray fluorescence (TXRF) analysis, showing Ir/M values of 0.9 and 1.4 for M = Sc and In, respectively, confirming a larger number of vacancies in the case of InIrPF-13. Nevertheless, the formation of an extended framework is ensured by the fact that at least one of the indium or scandium sites is always fully occupied. Thus, the connection of the two types of SBUs through the organic linkers result in the formation of a heterometallic corrugated layered coordination framework (Figure 1c). The layers are stacked, forming hydrogen bonds, generating oval-shaped channels, which are filled with water molecules (Figure 1d). The framework formula derived from the single crystal analysis is therefore [Ir_2_M_3–*x*_(2,5-PDC)_6_O_2–*x*_(H_2_O)_2_] where *x* is the number of vacancies at the indium or scandium centers. Elemental CHN analysis results are consistent with a formula where *x* = 1 (Calcd. for ScInPF-13 C, 33.3%; H, 1.47%; N, 5.55. Exptl. C, 33.2%; H, 1.76%; N, 5.77%. Calcd. for InIrPF-13 C, 30.4%; H, 1.34%; N, 5.07. Exptl. C, 30.0%; H, 2.20%; N, 4.9%).

**Figure 2:**
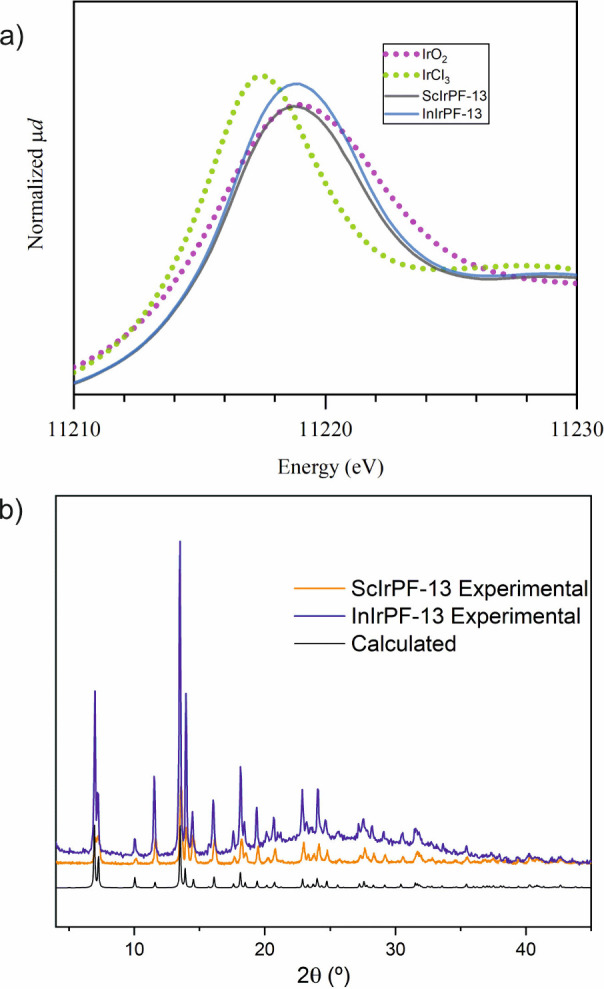
(a) Normalized X-ray absorption near-edge spectra for InIrPF-13 and ScIrPF-13, IrCl_3_, and IrO_2_. (b) Experimental and simulated PXRD patterns of MIrPF-13 (M = In, Sc).

Synchrotron X-ray absorption near edge spectroscopy (XANES) measurements were conducted for the MOF samples and for reference compounds IrCl_3_ and IrO_2_, to investigate the oxidation state of iridium atoms. The position of the absorption maximum for both Sc- and InIrPF-13 pellet samples was found to be at the same energy as that of IrO_2_, indicating that iridium atoms have been oxidized to Ir^4+^ during the MOF synthesis reaction (Figure 2a).

The powder X-ray diffraction (PXRD) patterns of the bulk samples demonstrated the formation of InIrPF-13 and ScIrPF-13 (Figure 2b). The thermal gravimetric analysis plot of ScIrPF-13 activated under vacuum overnight (Figure S5) displays one weight loss at around 60–100 °C, corresponding to the loss of water molecules adsorbed in the pores of the MOF, and the total decomposition of the framework occurs at 440 °C in only one step (400 °C for InIrPF-13). Regarding porosity, we found that after activation at 100 °C, ScIrPF-13 displays an N_2_ sorption isotherm profile characteristic of layered materials with intercalated molecules, and a BET surface area value of 24 m^2^/g (Figure S6).^50^ The stability of the new MOFs under acidic or basic conditions was also evaluated. The PXRD patterns of the recovered samples show that they maintain the same structure after being immersed for 24h in NaOH or nitric acid aqueous solutions, in the pH range 5–10. The sample remains crystalline at pH = 3, although small differences are appreciated in the PXRD pattern, possibly indicative of initial structural changes (Figure S7).

Upon successfully obtaining the novel iridium MOFs, we moved toward proving that the material can effectively be used as a heterogeneous catalyst and demonstrating the activity of the reticulated iridium sites. In particular, we decided to investigate the photocatalytic activity^51^ of the new MOF under visible light irradiation. Iridium complexes are receiving increasing attention as sensitizers in numerous light-induced photoredox catalytic transformations due to the broad range of potentials that they might exhibit through linker modification.^52−54^ As a demonstration of the suitability of ScIrPF-13 as a photoredox heterogeneous catalyst, we investigated its use in the photooxidation of sulfides, a reaction that has been proved to be catalyzed by other iridium containing materials,^55,56^ including MOFs where iridium atoms are anchored at the organic linker.^57^ Thus, in the presence of 2 mol % of the catalyst (based on Ir), the model substrate methylphenylsulfide was quantitatively and selectively oxidized to the corresponding sulfoxide under light blue irradiation (420 nm, Table 1, Figure S8 for UV–vis spectra), after 20 h under an O_2_ or air atmosphere. Remarkably, the catalyst was easily recovered and reused, with no significant loss of activity or selectivity observed after six consecutive cycles. Furthermore, the very slow decay in activity, averaging just 1% per cycle (Table 1), strongly suggests that the material can be effectively reused over a significant number of cycles. Moreover, the PXRD pattern of the recovered catalyst (Figure S9) demonstrates that the structure is preserved. The photocatalytic activity of the MOF was further evaluated with other substituted sulfides. Thus, *p*-tolylmethylsulfide and 4-chlorophenylmethylsulfide were also fully oxidized to their corresponding sulfoxides, after 28 and 36 h of reaction, respectively. We also tested the ability of ScIrPF-13 to photooxidize the toxic sulfur mustard simulant 2-(chloroethyl)ethylsulfide,^58^ again obtaining quantitatively the less toxic sulfoxide in 20 h. Moreover, ScIrPF-13, also demonstrated to be active in other photocatalyzed processes, such as oxidation of alcohols to aldehydes (55% conversion of (4-methoxyphenyl)methanol to 4-methoxybenzaldehyde) or hydroxylation of phenylboronic acid to phenol (55% conversion; Table S5).

**Table 1:**
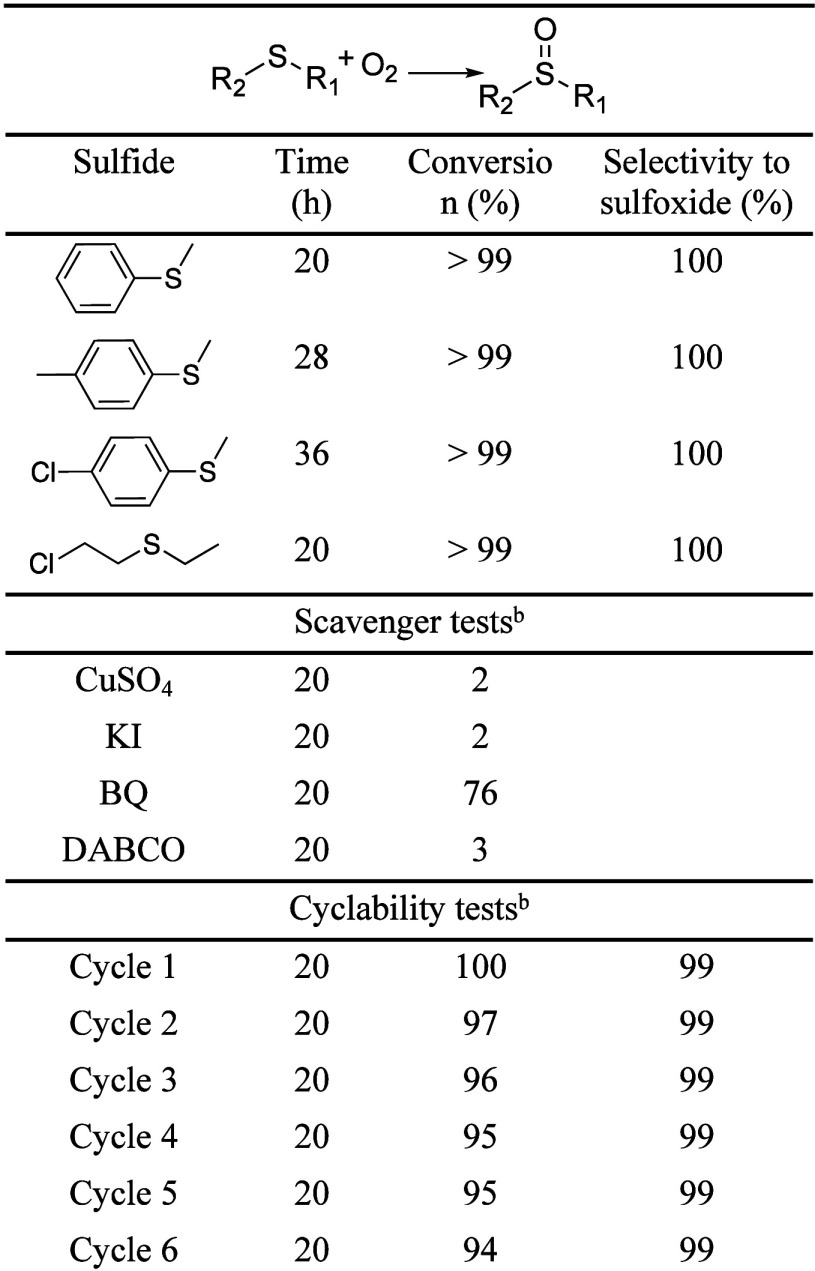
Photooxidation of Sulfides with ScIrPF-13^a^

a Photooxidation conditions: sulfide (0.135 mmol), catalyst (2 mol% Ir), acetonitrile (0.5 mL), irradiated with blue LED light (420 nm, 2 × 30W) at RT under O_2_ balloon atmosphere.

b Carried out with methylphenylsulfide.

**Figure 3:**
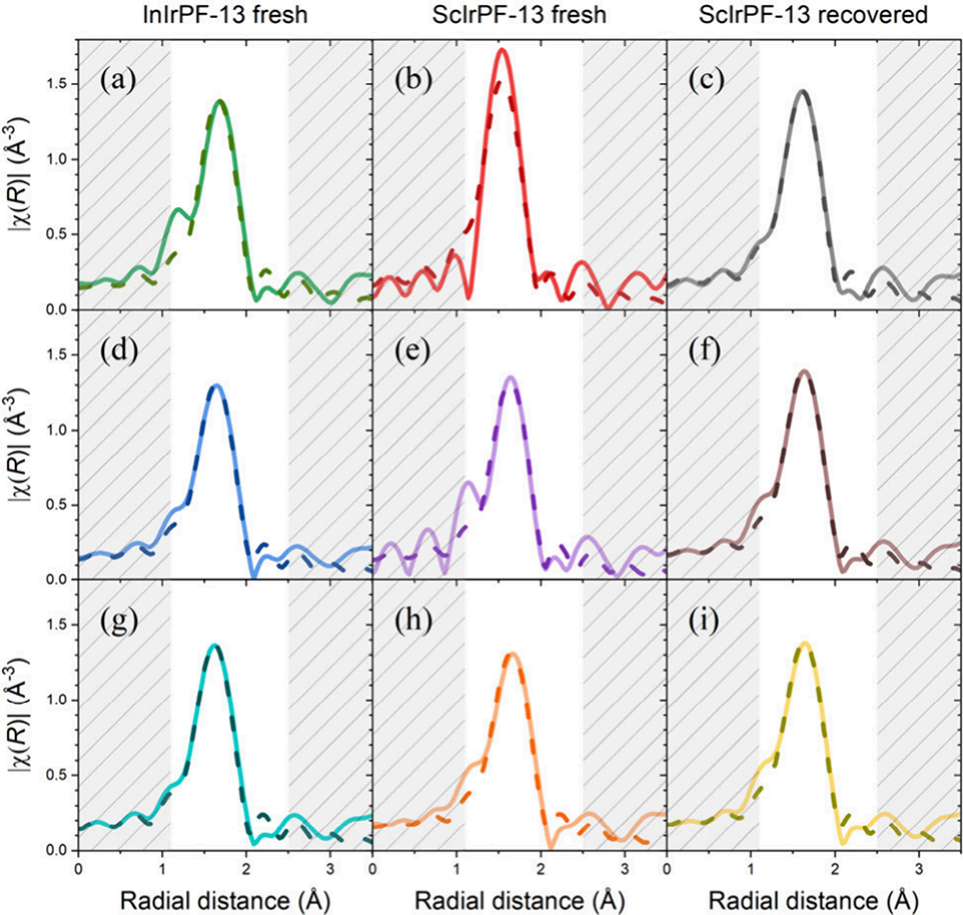
Fourier transform of the k2-weighted EXAFS spectra (solid lines) and fitting results (dashed lines) at the Ir L3-edge for fresh InIrPF-13 (a, d, g) and ScIrPF-13 (b, e, h) and recovered ScIrPF-13 (c, f, i) samples. Data were collected for the pellets in air (a–c), under light irradiation (d–f), and after switching off the light (g–i).

Control experiments were carried out in the absence of iridium, by using a previously reported MOF composed of scandium and the same organic linker.^59^ As expected, this MOF did not show any activity under the same reaction conditions, demonstrating the need of the iridium atoms to catalyze the oxidation process. To get further insights on the role of the iridium atoms during the reaction and the possible interactions with the substrates, we also completed an extended X-ray absorption fine structure (EXAFS) analysis for pellet samples of freshly prepared MOFs and recovered from the catalytic test. Measurements were made in air, under the same illumination conditions, with and without light (Figure 3). The fitted parameters indicate that no changes are produced in the coordination environment of the iridium atoms due to the illumination process or after interaction with the catalytic substrates (Table S6). To ensure that no changes in the Ir environment take place during the reaction, measurements were also completed for the ScIrPF-13 sample suspended in acetonitrile inside a glass pipet and in the presence of methylphenyl-sulfide under illumination for 15 h. The evolution of interatomic distances and coordination number of the first-shell over time were obtained by fitting Fourier transformed (FT) EXAFS spectra. Both parameters fluctuated around the same values, and only small changes in the intensity of the white line were observed, which are explained by fluctuations in the amount of material interacting with the beam, as indicated by the result of the multicomponent analysis (MCR) of the XANES spectra (Figures S10–S12). Notably, the oxidation state of Ir remains +4 throughout the entire reaction. These results indicate that the reaction does not require direct interaction of the substrate molecules in the first coordination sphere of the iridium atoms and that this was maintained unaltered after the catalytic process. The reaction must, therefore, occur through the reactive oxygen species (ROS) generated after the light-initiated charge separation process in ScIrPF-13.

To investigate the nature of the ROS involved in the photooxidation process, reactions were carried out in the presence of different scavenger species. In particular, when in the presence of *p*-benzoquinone (BQ), more than 75% conversion was achieved, ruling out the superoxide anion as the main ROS. On the contrary, when 1,4-diazabicyclo[2.2.2]octane (DABCO) was present, the conversion drastically dropped to less than 5%, clearly indicating that ^1^O_2_ is the most important ROS generated upon energy transfer from the MOF.^60^ Indeed, by transient measurements, we observe that, in the presence of molecular oxygen, the MOF is already quenched (Figure S13). Moreover, the presence of electron and hole scavengers, namely, copper sulfate and potassium iodide, respectively, also resulted in drastic inhibition of the reaction (<5% conversion), indicating the critical role of the charge separation process initiated by ScIrPF-13 in the photooxidation process.

**Figure 4:**
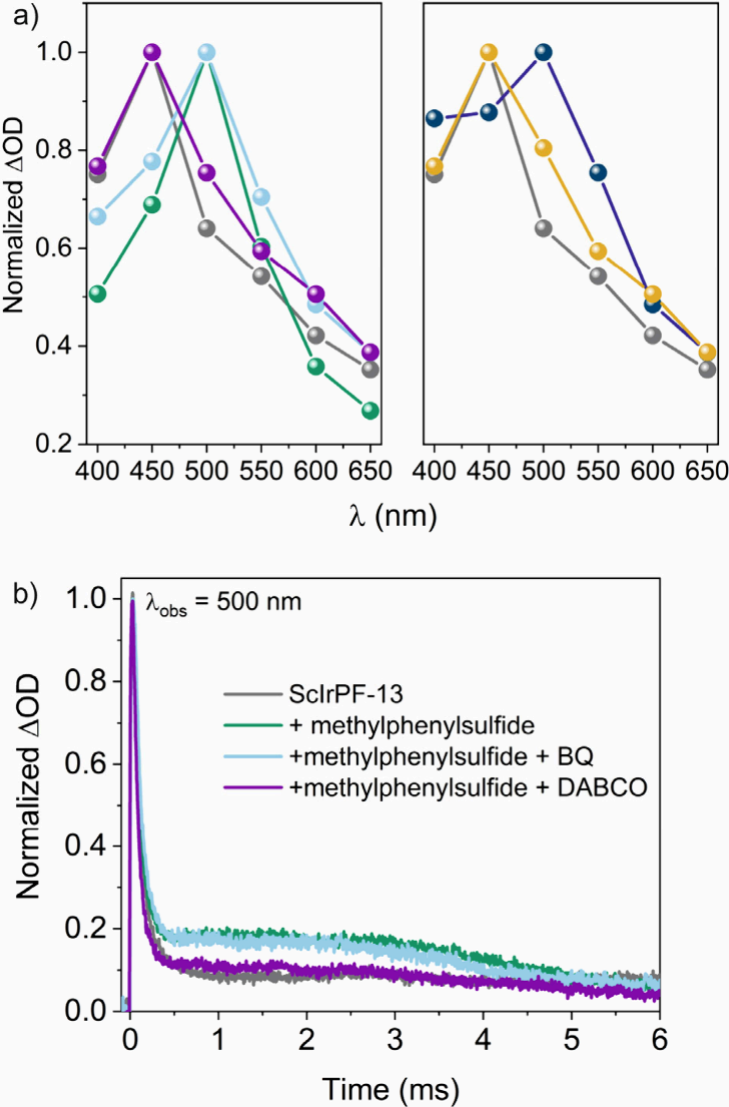
(a) Transient absorption spectra (TAS) for ScIrPF-13 (gray), and in the presence of methylphenylsulfide (green), methylphenylsulfide + BQ (cyan), or methylphenylsulfide + DABCO (purple), and in the presence of CuSO_4_ (dark blue) or KI (orange). (b) Corresponding transient decay traces (λ_exc_ = 355 or 410 nm, λ_obs_ = 500 nm). All measurements were performed in acetonitrile dispersion solutions under an air atmosphere.

Further experiments were then performed by means of transient absorption spectroscopy (TAS). Thus, after laser excitation, bare ScIrPF-13 exhibited a TA spectrum covering all spectral windows with a maximum at 450 nm (Figure 4, gray trace, and Figure S14). However, in the presence of methylphenylsulfide, a new transient band centered at 500 nm was observed (Figure 4, green trace, and Figure S14), which could be ascribed to the formation of the intermediate from the corresponding aromatic sulfide previous to the final product formation.^61,62^ Then, scavenger tests were carried out, and interestingly, the 500 nm TA band showed no changes in the presence of BQ, while the presence of DABCO resulted in TA spectra mainly identical to that obtained for the bare ScIrPF-13, avoiding the formation of the 500 nm transient (Figure 4, blue and purple traces, and Figure S14). These results support the main role of ^1^O_2_ in the ScIrPF-13 initiated photocatalytic process.

In terms of transient lifetimes, bare ScIrPF-13 was fitted following a three-exponential function resulting in lifetimes (τ) of 89, 148, and 9086 ns, respectively (calculated average lifetime was of 8.05 μs). The addition of methylphenylsulfide resulted in an increment of double the ΔOD (between 0.2 and 5 μs time scale) by formation of the 500-nm TA band. As previously observed in Figure 4a, upon addition of BQ, the signal remained unaltered while the presence of DABCO resulted in a decrease in τ up to obtaining an identical lifetime to that of the naked MOF (Figure 4b). Control experiments in the absence of ScIrPF-13 resulted in a null interaction between methylphenylsulfide with BQ or DABCO (Figure S15).

On the other hand, the addition of CuSO_4_ or KI revealed the nature of the photogenerated holes (h^+^) or electrons (e^–^) in ScIrPF-13, respectively (Figures 4 and S16). Photohole absorption resulted in a broad peak between 450 and 550 nm, whereas photoelectrons were also monitored exhibiting a broad peak between 400 and 500 nm. All TAS results were in agreement with the results of the photocatalytic tests employing scavengers as shown in Table 1, clearly demonstrating the main role of iridium atoms to induce efficient electron–hole separation (Figure 4), to produce the photooxidation reaction.

## Conclusions

In summary, we have demonstrated here, for the first time, the feasibility of synthesizing metal–organic frameworks with iridium as a structural chemical building component in a one-synthesis step. Furthermore, iridium atoms are oxidized during the synthesis reaction, being a unique example of the incorporation of Ir^4+^ in a MOF, and moreover their oxidation state and coordination environment remain unaltered after their use as heterogeneous photocatalysts. Our multimetal-based approach paves the way to extend the field of reticular chemistry to previously unexplored metal elements, incorporating new active metal sites and expanding the range of reactivity achievable with MOFs. We anticipate that this approach will be instrumental in obtaining other frameworks with diverse chemical and pore environments specifically tailored to exploit the activity of these high-value metal elements in the future.

## Supporting Information Available

Additional experimental details, materials, and methods (PDF)

## Accession Codes

Crystal structures of InIrPF-13 and ScIrPF-13 have been deposited in the CCDC database with numbers 2304062 and 2304063.
