# Supplementary material for: A Multimetal Approach for the Reticulation of Iridium into Metal–Organic Framework Building Units
Source: J Am Chem Soc. 2024 Sep 4;146(37):25824–31. doi: 10.1021/jacs.4c08638 (PMC11421005; doi:10.1021/jacs.4c08638)
Supplement: Supplementary file 1 — ja4c08638_si_001.pdf [file ja4c08638_si_001.pdf]

Supporting Information

A Multi-metal Approach for the Reticulation of Iridium into Metal-Organic Framework Building Units

Raluca Loredana Vasile,[a] M. Carmen Borrallo-Aniceto,[a] Fátima Esteban-Betegón,[a] Alina A. Skorynina,[b] Miguel Gomez-Mendoza,[c] Victor A de la Peña O'Shea,[c] Enrique Gutiérrez Puebla,[a] Marta Iglesias,[a] M. Ángeles Monge,[a] Felipe Gándara.[a]*

[a]Materials Science Institute of Madrid – Spanish National Research Council (ICMM-CSIC). Calle Sor Juana Inés de la Cruz 3, 28049, Madrid, Spain.

[b]CELLS-ALBA Synchrotron Radiation Facility, 08290, Barcelona, Spain

[c]Photoactivated Processes Unit, IMDEA Energy Institute, Ramón de la Sagra 3, 28935, Móstoles, Spain

***gandara@icmm.csic.es**

Materials and Methods

S1.1 Synthesis of MIrPF-13 (M = In, Sc)

All reagents and solvents employed were commercially available and used as received without further purification: 2,5-pyridinedicarboxilic acid, 2,5-H_2PDC (>98%, TCI); iridium(III) chloride hydrate, $IrCl_3 \times nH_2O$ (99.8%, Alfa Aesar); indium(III) acetate, $In(CH_3COO)_3$ (99.99%, Alfa Aesar); scandium nitrate pentahydrate, $Sc(NO_3)_3 \times 5H_2O$ (99.9%, Strem Chemicals); indium(III) nitrate hexahydrate, $In(NO_3)_3 \times 6H_2O$ (99.99%, Alfa Aesar); indium(III) chloride tetrahydrate, $InCl_3 \times 4H_2O$ (99.99%, Strem); indium(III) fluoride hydrate, $IrF_3 \times nH_2O$ (99%, abcr); indium(III) acetylacetonate, $In(acac)_3$ (≥99%, Sigma Aldrich).

2,5- H_2PDC (0.32 mmol, 54.6 mg), $Sc(NO_3)_3 \times 5H_2O$ (0.08 mmol, 25.7 mg) or $In(NO_3)_3$ (0.08 mmol, 23.4 mg), and $IrCl_3 \times nH_2O$ (0.08 mmol, 24.0 mg) were dissolved in 13 mL of deionized water. The ratio between the linker, $Sc(NO_3)_3 \times 5H_2O$ and $IrCl_3 \times nH_2O$ is 4:1:1. The mixture was placed in a 50 mL Teflon-lined steel autoclave and heated during 24 hours in an oven at 170 °C for ScIrPF-13, or during 10 days at 150 ºC for InIrPF-13. After cooling to room temperature, the yellow crystals were washed with water (3x10 mL).

During the synthesis optimization process for InIrPF-13, we noticed the presence of additional diffraction peaks that were identified as belonging to a previously reported indium MOF with the same linker, $[In(OH)(2,5\text{-}PDC)]_n$ (CCDC number 659856).[1] The presence of this phase, even as a minor impurity, was detected for a wide range of conditions, as detailed in tables S3 and S4, and could be avoided by increasing the reaction time at 150 ºC heating temperature, with nitrate as indium salt.

S1.2. Catalytic Procedure

All reagents and solvents employed were commercially available and used as received without further purification. Prior to the reaction, the catalyst was activated by heating it at 100 ºC for a overnight period. In a SUPELCO glass microreactor, 0.135 mmol of the corresponding substrate, 2 mol% of ScIrPF-13 catalyst (based on Ir), and 0.5 mL of acetonitrile were mixed. The resulting suspension was stirred under O_2 atmosphere (balloon), or air, and irradiated with 2 blue LED light (420 nm) 30 W lamps. The reactions were monitored by GC chromatography.

S1.3. Elemental Analysis (EA)

The carbon, nitrogen, and hydrogen content of the samples were determined by elemental analysis. The samples were vacuum-dried and sent to the EA department at the Chemical Analysis facility at the Materials Science Institute of Madrid (ICMM), where they operate a CNHS Perkin Elmer 2400 elemental analyzer.

S1.4. Thermogravimetric Analysis (TGA)

The thermal stability of the samples was determined by thermogravimetric analysis. The samples were sent to the TGA department at the Chemical Analysis facility at the Materials Science Institute of Madrid (ICMM), where they operate a simultaneous TGA/ATD STD-Q600 equipment from TA Instruments. The samples were placed in a platinum crucible, and heated in air (100 mL/min flow), in a temperature range between 25 °C and 800 °C with a rate of 10 °C/min.

S1.5. Infrared Spectroscopy (IR)

The samples were sent to the IR Spectroscopy and Ellipsometry facility at the Materials Science Institute of Madrid (ICMM), where they operate a Bruker Vertex 70V. The IR spectrum of the samples were recorded from KBr pellets in a range from 300 to 4000 cm^{-1}.

S1.6. Powder X-Ray Diffraction (PXRD)

Powder X-Ray Diffraction patterns were collected at the Diffraction Facility of the Materials Science Institute of Madrid (ICMM), with a Bruker D8 DaVinci diffractometer equipped with a CuKα radiation tube ($K\alpha_1$ = 1.5406 Å, $K\alpha_2$ = 1.5444 Å, $K\alpha_1/K\alpha_2$ = 0.5) operated at a voltage of 40 kV and at a current of 40 mA. Each experiment was recorded with an exposure time of 0.1 seconds per step, and a step size of 0.02°. The samples were prepared by placing a small amount of a suspension of crystals in acetone on a glass sample carrier, forming a thin layer in the center.

S1.7. Single-Crystal X-Ray Diffraction (SCXRD)

Single-crystal X-Ray Diffraction data was collected at the Diffraction Facility of the Materials Science Institute of Madrid (ICMM). The samples were placed on a glass slide and submerged in mineral oil. Suitable crystals were selected with a kapton loop from MiTeGen, using a polarized optical microscope to check their size and quality. Diffraction data was collected with a Bruker D8 Venture diffractometer, equipped with three microsources (Cu, Mo, Ag) and a PHOTON III detector.

S1.8. Scanning Electron Microscopy (SEM) and Energy Dispersive X-Ray Spectroscopy (EDX)

Scanning Electron Microscopy studies were conducted at the Microscopy facility of the Materials Science Institute of Madrid (ICMM), with a FE-SEM FEI Nova NANOSEM 230 microscope equipped with an Everhard-Thornley ETD detector and an operating voltage between 5 and 15 kV. The samples were prepared by placing the crystals on a double-sided adhesive conductive carbon tape that was attached to a flat aluminum sample holder, which was metallized with a gold layer of 127.5 Å with a Leica EM ACE200 sputter. EDS microanalyses were performed with an EDAX Apollo 10-300 mm detector. Several points of the crystals were recorded, generally from the basal planes and the body of the crystal.

S1.9. Flame Ionization Detector-Gas Chromatography (FID-GC)

FID-GC measurements were conducted at the Institute of Materials Sciences of Madrid (ICMM), with a Konik 5000C chromatography system, equipped with a flame ionization detector. Mesitylene was used as a standard in a 1:1 ratio with the substrates.

S1.10. X-ray Absortion spectroscopy

X-ray absorption spectra (XAS) at the iridium L_3-edge were recorded at the CLAESS beamline of the ALBA Synchrotron, Barcelona, Spain. Data was acquired at room temperature in transmission mode using a Si311 double crystal monochromator. The beam was focused horizontally and vertically using a toroidal mirror coated with Rh. Ex situ samples were prepared as pellets of 13 mm diameter. For in situ experiment powder catalyst ScIrPF-13 was loaded in a glass pipette with reagents in air and placed between two 30W light sources. The spectra collected ex situ were calibrated, aligned and normalized with background removal using the Athena software package.[2] For in situ experiment XAS spectra were processed in Python 3.7 using pyMCR library for

principle component analysis (PCA) and multivariate curve resolution analysis[3] of XANES spectra, and Larch library[4] for EXAFS fitting.

S1.11. Transient absorption spectroscopy (TAS)

Measurements were carried out with a laser flash photolysis LP980 equipment from Edinburgh Instruments, based on an optical parametric oscillator (OPO) pumped by a Nd:YAG laser (EKSPLA). The selected excitation wavelength for the measurements was the third harmonic of the laser (355 nm) or 420 nm using the OPO mode. The same results were obtained using both excitation wavelengths. The corresponding single laser pulses were of 1 mJ per pulse of ≈5 ns duration, while a pulsed xenon flash lamp (150 W) was employed as detecting light source. The probe light is dispersed through a monochromator (TMS302-A, grating 150 lines mm−1) after it has passed the sample and then reaches a PMT detector to obtain the temporal profile. The absorbance of all samples was kept at ≈ 0.1 at λ excitation as dispersed solutions in acetonitrile as solvent. Measurements were conducted using scavengers to simulate the reaction media in photocatalytic studies. The scavengers employed were: a) methylphenylsulfide as model substrate (10 mM), *p*-benzoquinone (BQ, 32 μM), 1,4-diazabicyclo[2.2.2]octane (DABCO, 45 μM), copper sulfate ($CuSO_4$, 45 μM) and potassium iodide (KI, 45 μM). The transient spectra (Figure 4) were obtained by taking the maximum absorption value (ΔOD) for each experimental decay trace (measured from 400 to 650 nm each 50 nm). All transient spectra were recorded at room temperature using 10 × 10 mm^2 quartz cells, in aerated conditions.

S1.12. Total Reflexion X-ray fluorescence (TXRF)

Measurements were carried out at the Autonomous University of Madrid, with a Bruker S2 PicoFox spectrometer, using Mo radiation as excitation source, with a 50 kV voltage, and 600 μA current, and using vanadium as internal standard for the quantification of metal content.

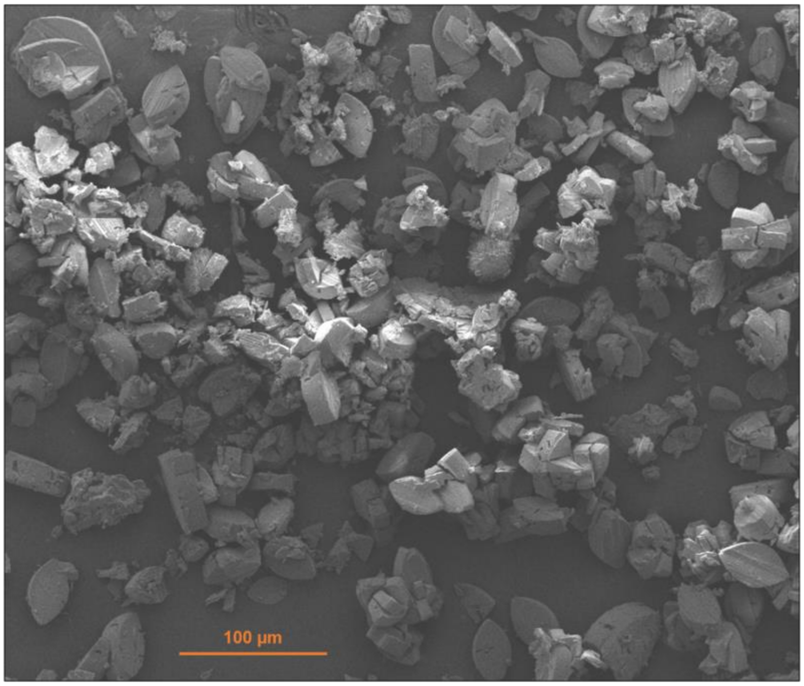

Figure S1. SEM image of the bulk of the sample ScIrPF-13.

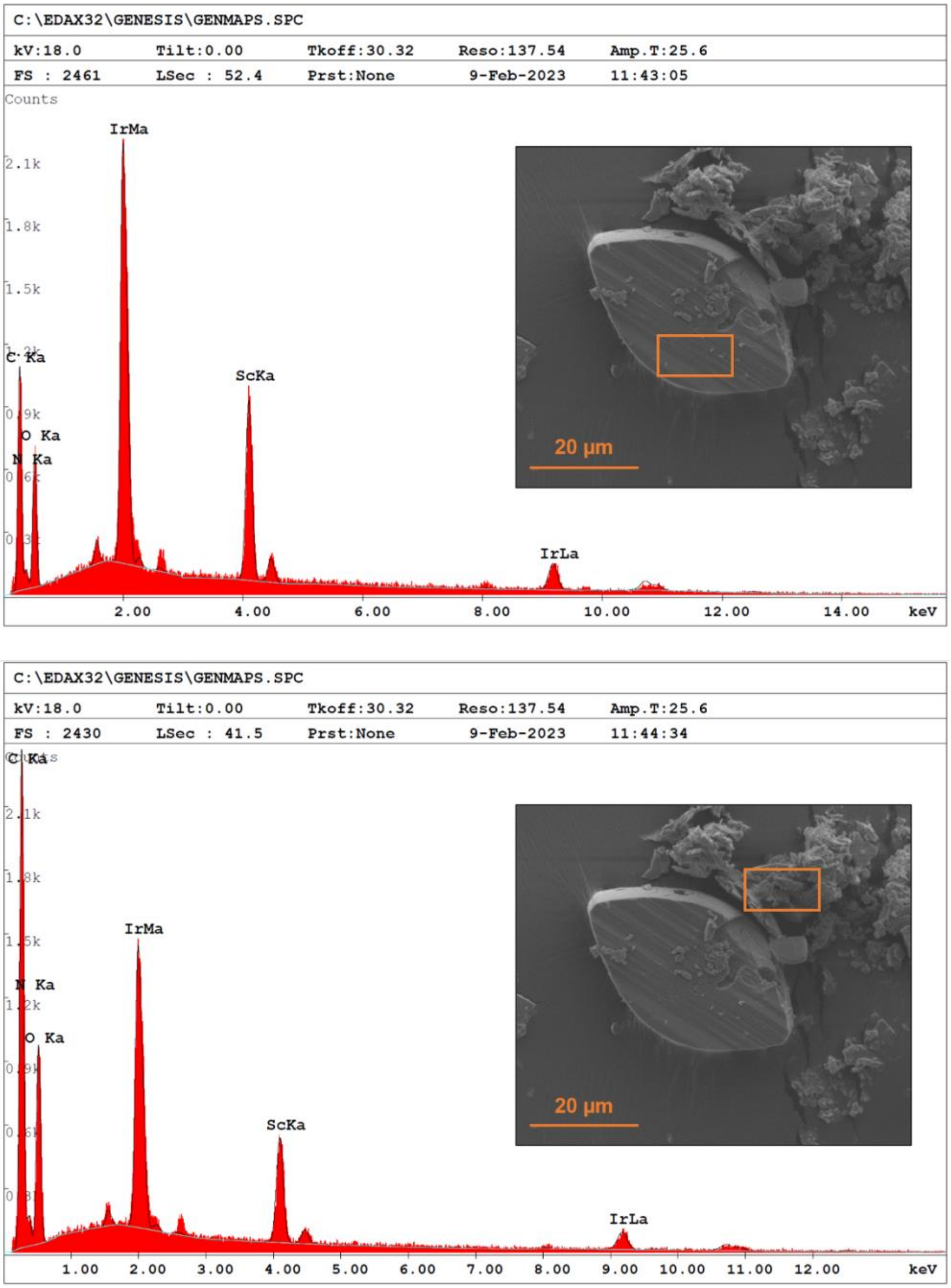

Figure S2. EDX spectrum of an individual crystal **(up)** and an aggregate of particles of ScIrPF-13 **(down)**. In both cases, the ratio between Sc and Ir is 1.3.

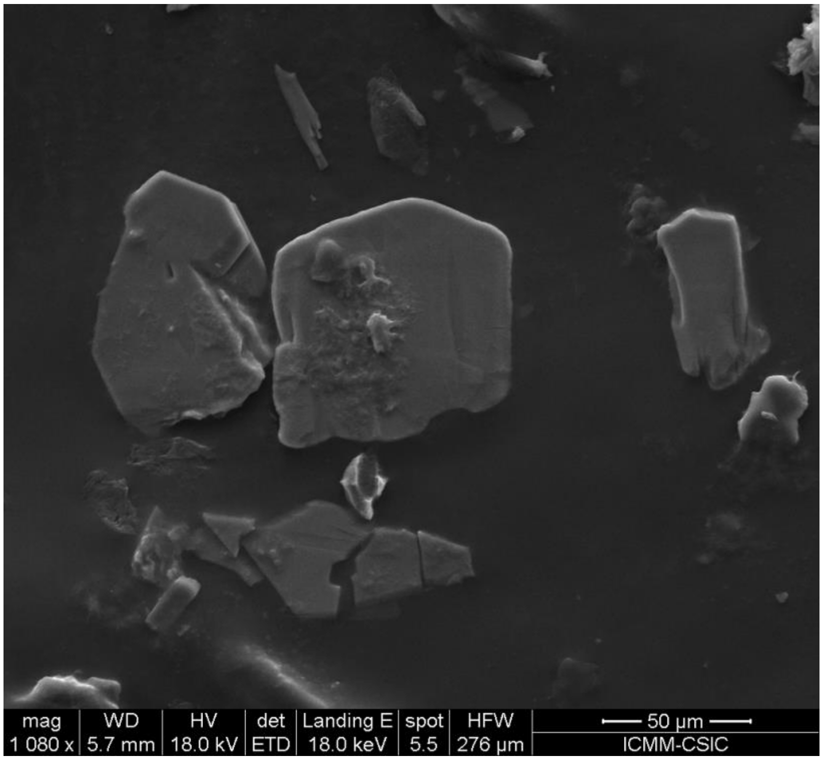

Figure S3. SEM image of the bulk of the sample InIrPF-13.

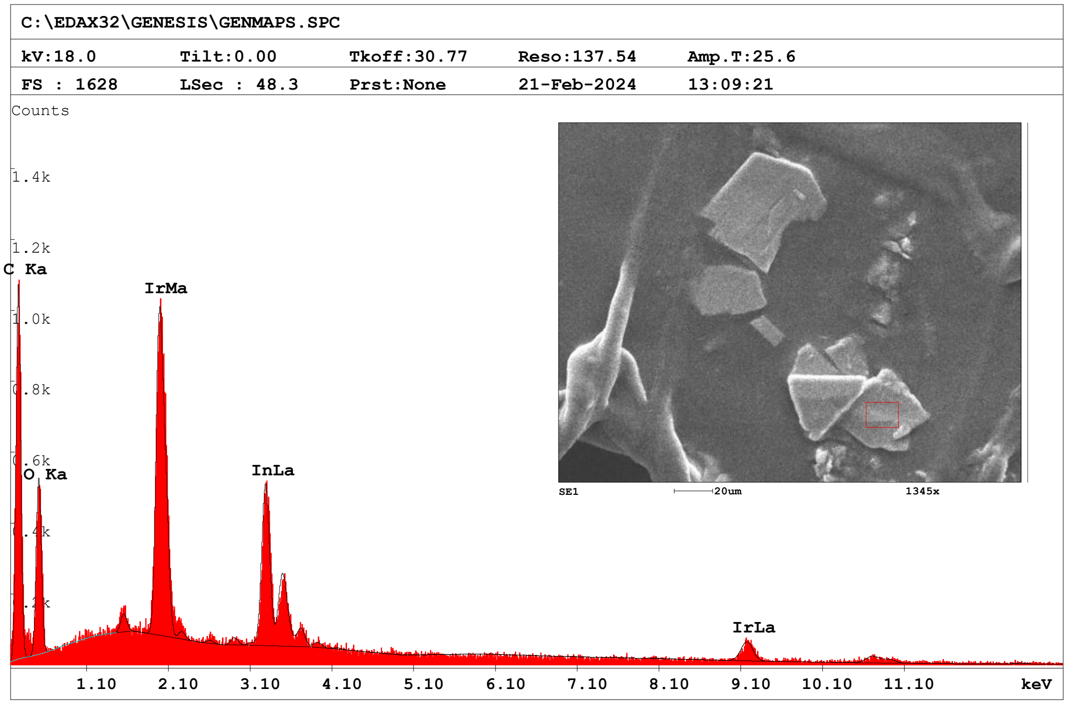

Figure S4. EDX spectrum of InIrPF-13 crystal indicating a ratio between In and Ir of 1.1.

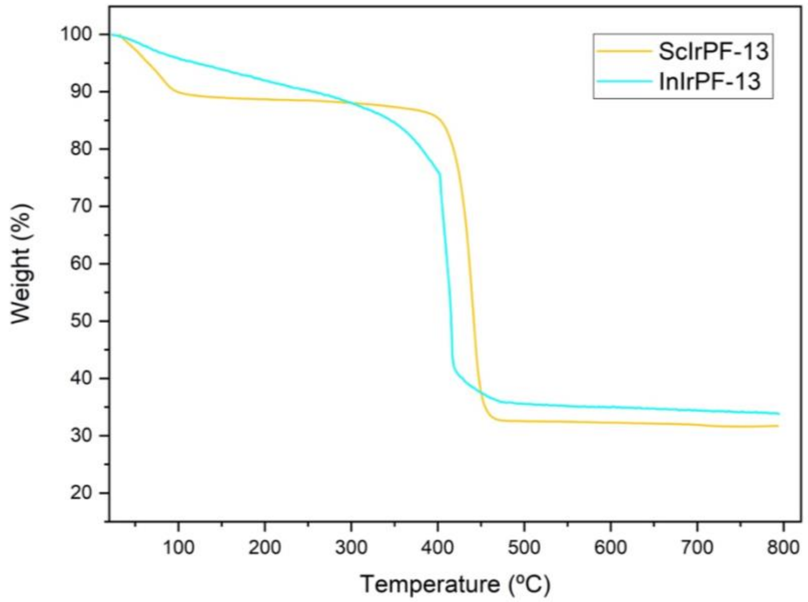

Figure S5. TGA plot of ScIrPF-13 and InIrPF-13 showing a weight loss of 11 % around 60-100 ºC, corresponding to the loss of solvent molecules (water) adsorbed in the pores of the MOF, and the total decomposition of the sample that occurs at 440 ºC (ScIrPF-13) or 400 ºC (InIrPF-13) in only one step which is equivalent to a weight loss of 56 % or 50% for ScIrPF-13 and InIrPF-13, respectively.

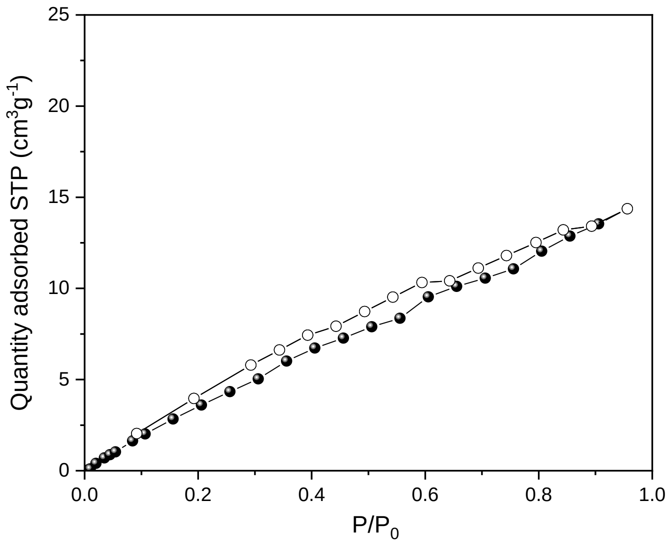

Figure S6. Adsorption-desorption isotherm of ScIrPF-13 activated at 100 ºC. Filled and hollow circles represent adsorption and desorption points, respectively.

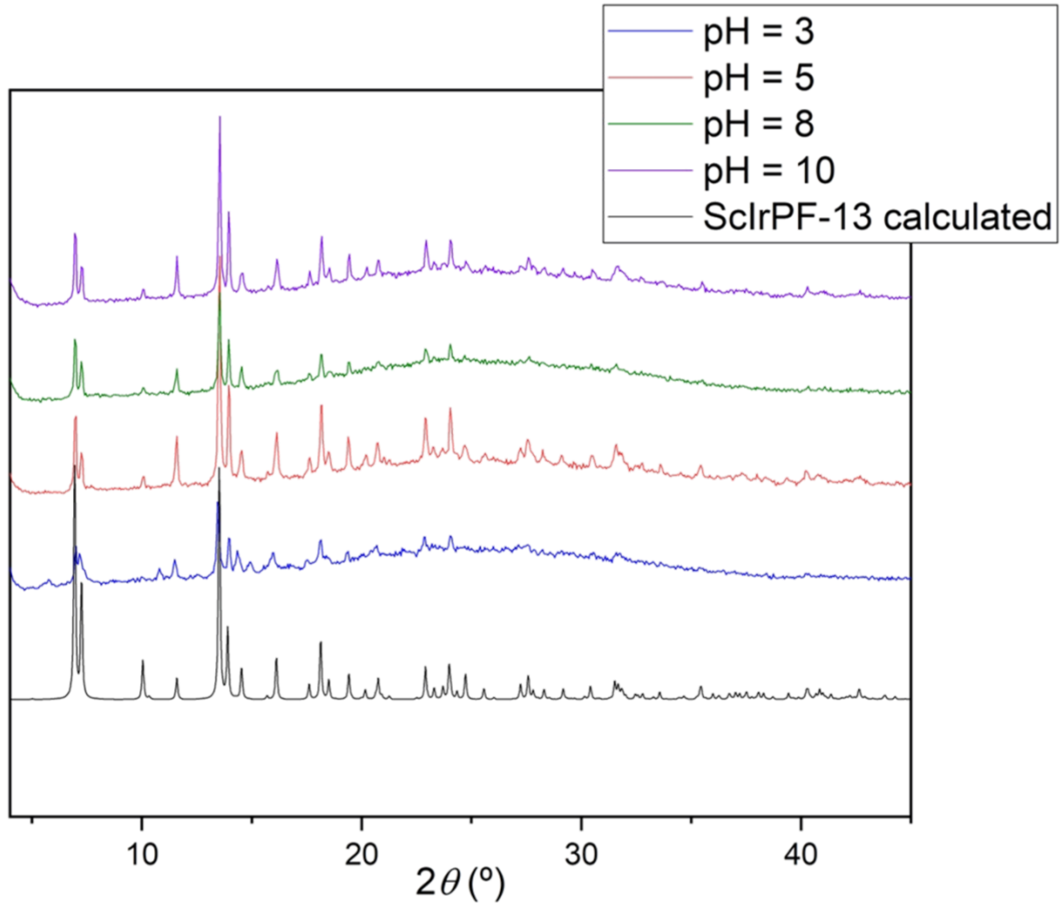

Figure S7. PXRD patterns of ScIrPF-13 after being immersed in NaOH or HNO_3 aqueous solution for 24 hours.

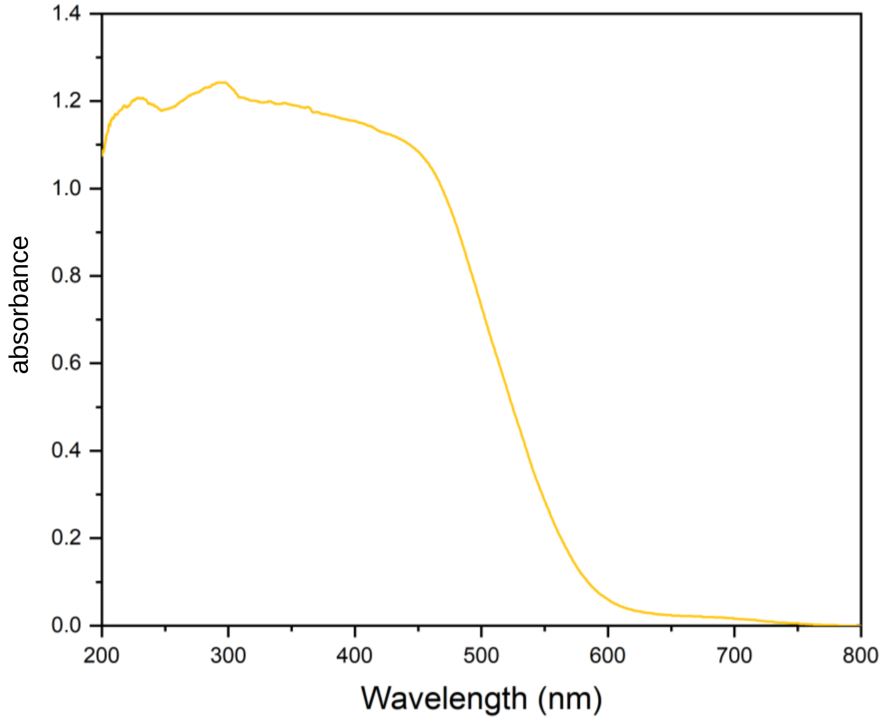

Figure S8. UV-Vis absorbance spectrum of ScIrPF-13.

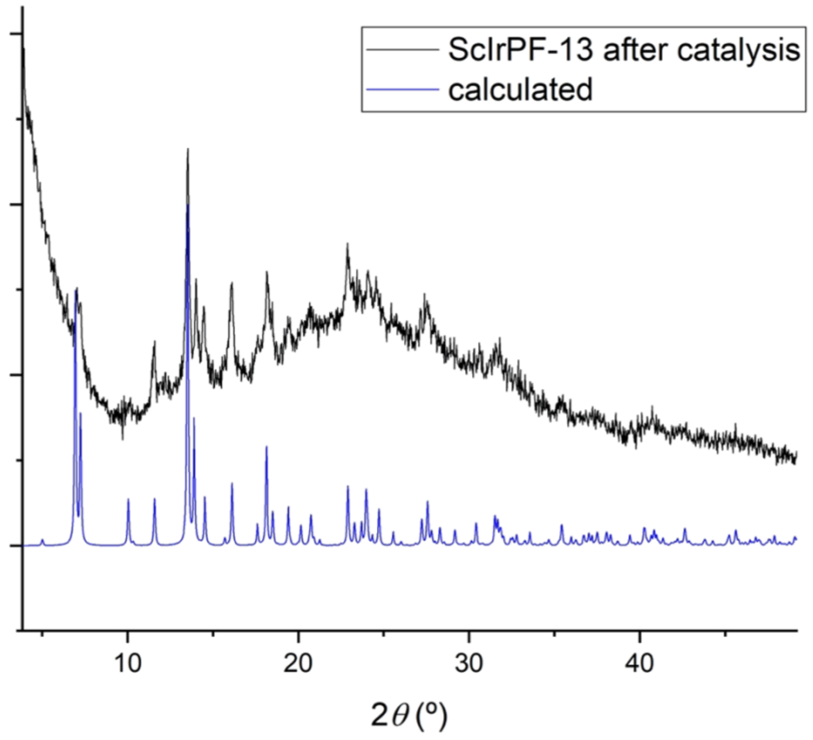

Figure S9. Experimental PXRD pattern of ScIrPF-13 recovered after catalytic reaction, compared with pattern calculated from single crystal data.

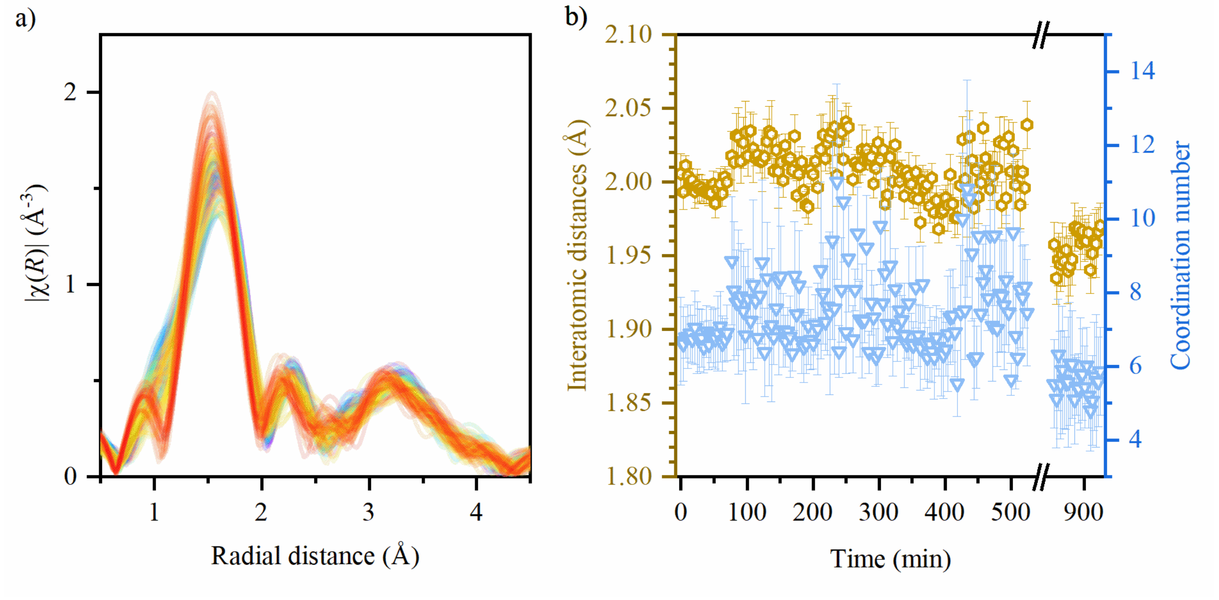

Figure S10. (a) Fourier transform of the k^2-weighted EXAFS spectra at the Ir L_3-edge for the ScIrPF-13 sample immersed in acetonitrile and in presence of methylphenyl-sulfide irradiated with visible light. (b) Ir–N/O interatomic distances and coordination numbers determined by first-shell fitting of EXAFS data of the part a using the Larch library.

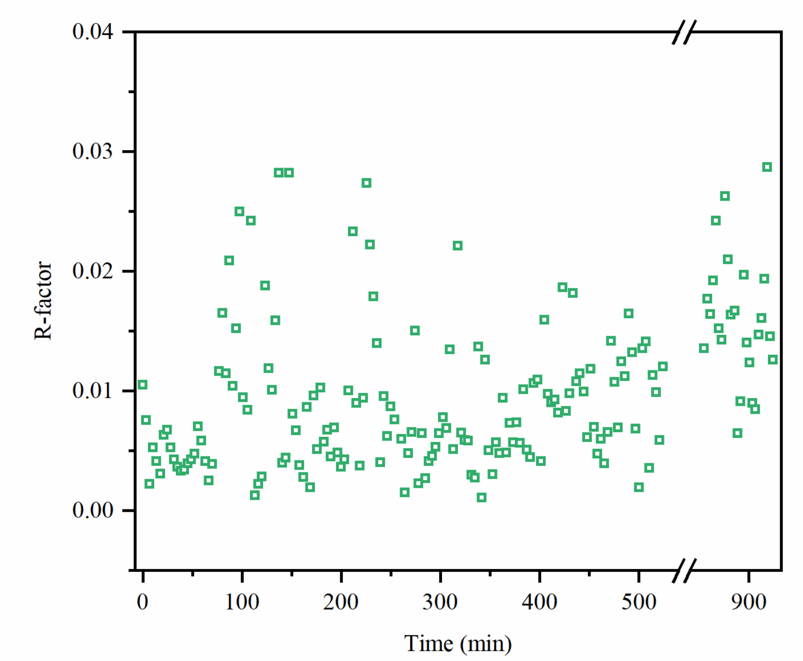

Figure S11. The R-factor of EXAFS fitting for the ScIrPF-13 sample immersed in acetonitrile and in the presence of methylphenyl sulfide, irradiated with visible light, fitted using the Larch library.

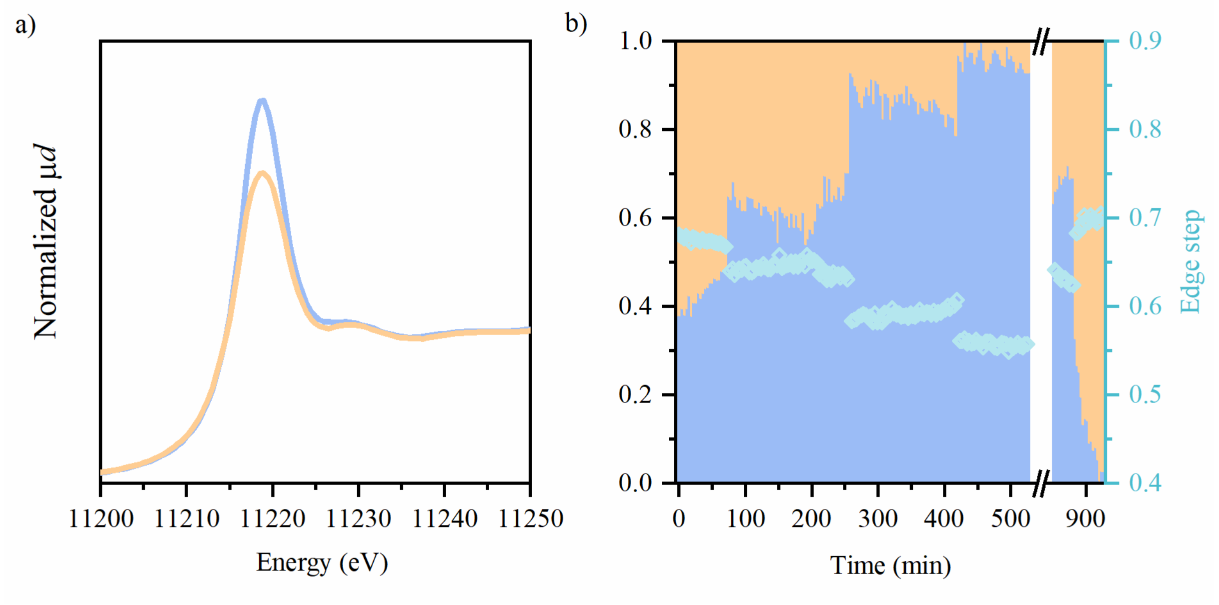

Figure S12. (a) Ir L_3-edge XANES spectra extracted from the whole in situ experimental dataset by MCR approach. (b) Evolution of MCR components (the color code is the same as for spectra presented in part a) and change of edge step with time.

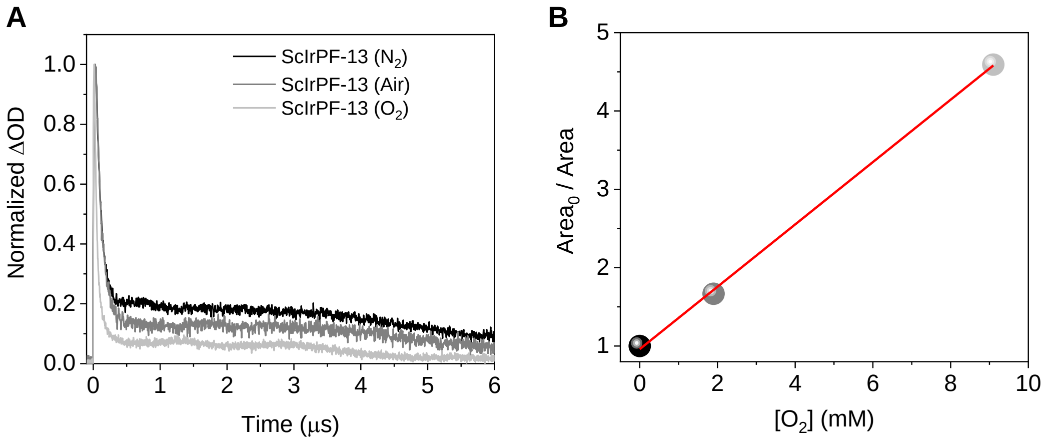

Figure S13. A) Normalized transient decay traces for **ScIrPF-13** in acetonitrile in aerated and purged (by N_2 or O_2) atmosphere. B) Corresponding Stern-Volmer relationship with relative area (A_0/A) *versus* O_2 concentration. It has been taken into account the area value (A) due to the multicomponent nature of the decay.

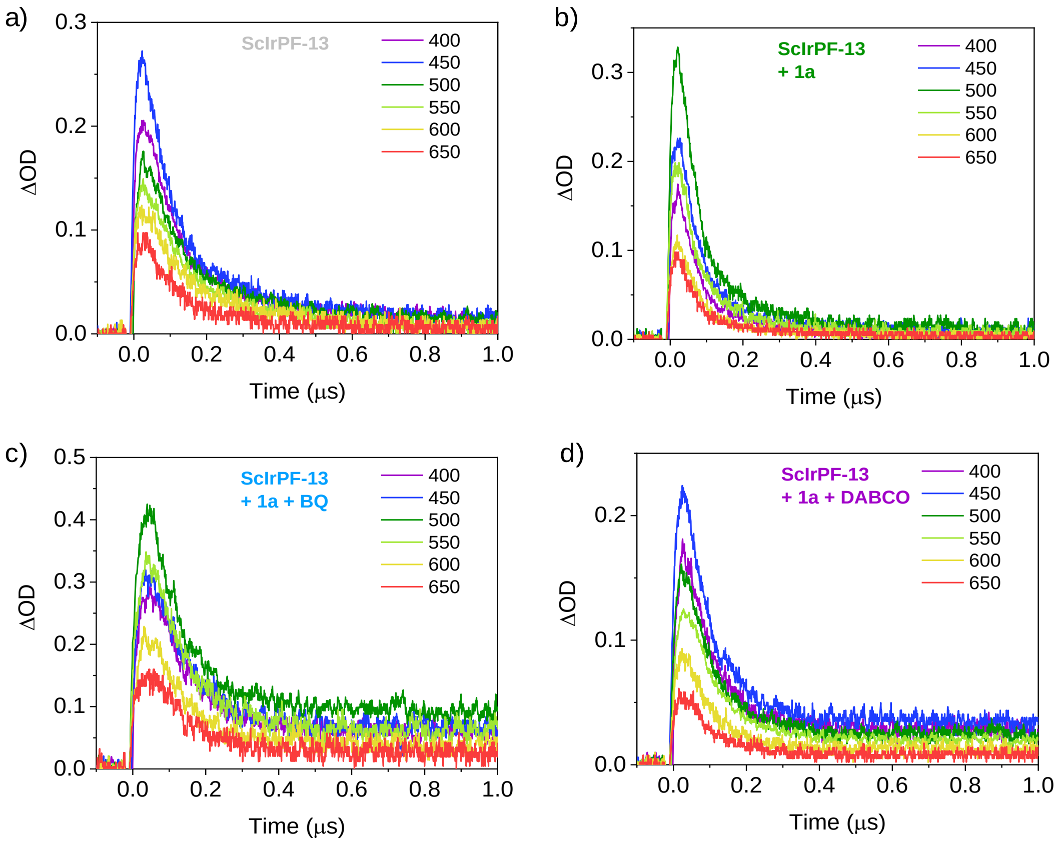

Figure S14. Consecutive transient decay traces (λ_{exc} = 355 or 410 nm) in the range of 400-650 nm in aerated acetonitrile dispersions for a) ScIrPF-13, b) ScIrPF-13 + 1a, b) ScIrPF-13 + 1a + BQ and d) ScIrPF-13 + 1a + DABCO, where 1a = mehtylphenylsulfide.

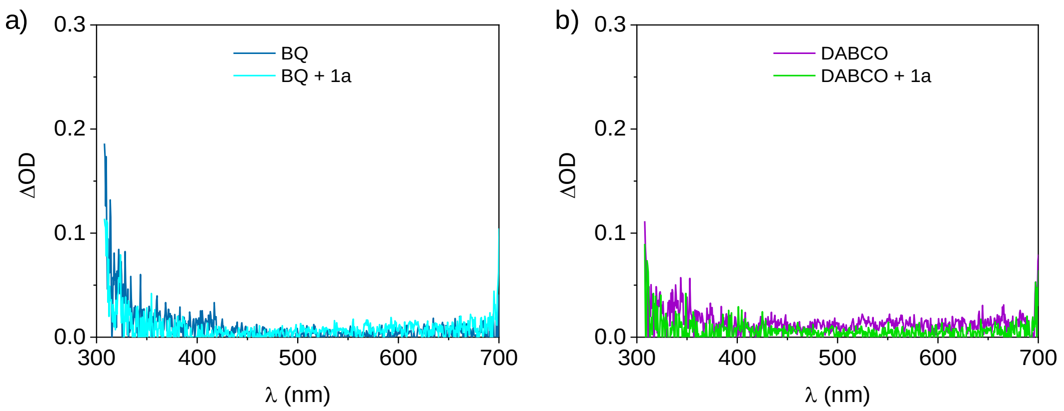

Figure S15. Transient decay spectra (λ_{exc} = 355 or 410 nm) for a) BQ (●) or b) DABCO (●) and its corresponding comparison in a sample mixture with 1a (● and ●, respectively) (1a = methylphenylsulfide).

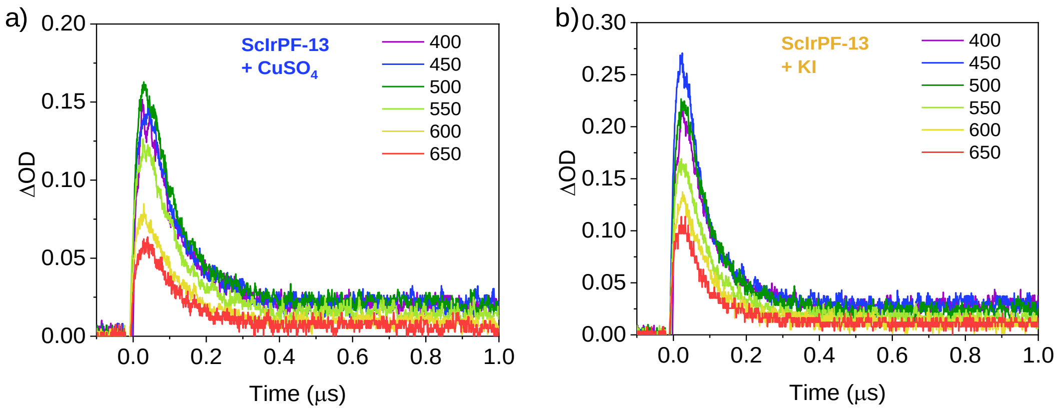

Figure S16. Consecutive transient decay traces (λ_{exc} = 355 or 410 nm) in the range of 400-650 nm in aerated acetonitrile dispersions for a) ScIrPF-13 + $CuSO_4$ and b) ScIrPF-13 + KI.

Table S1. Crystallographic parameters and refinement data for InIrPF-13.

Crystallographic parameters	InIrPF-13
Empirical formula	$C_{42}H_{18}Cl_{0.5}In_{2.21}Ir_{2}N_{6}O_{28.21}$
Formula weight (g/mol)	1713.86
Crystal system	Orthorhombic
Space group	$Cmc2_1$
a (Å)	25.6795(12)
b (Å)	23.9298(11)
c (Å)	9.8202(5)
α (°)	90
β (°)	90
γ (°)	90
Volume (Å^3)	6034.6(5)
Z	4
Calculated density (g/cm^3)	1.886
Absorption coefficient (mm^{-1})	15.959
F(000)	3234.0
Radiation	CuKα (λ = 1.54178)
2θ range for data collection (°)	5.048 to 130.204
Index ranges	$-30 \leq h \leq 27$, $-25 \leq k \leq 28$, $-11 \leq l \leq 11$
Reflections collected	27820
Independent reflections	5197 [Rint = 0.0309, Rsigma = 0.0218]
Data/restraints/parameters	5197/0/406
Goodness-of-fit on F^2	1.056
Final R indexes [I>2σ (I)]	R1 = 0.0300, wR2 = 0.0798
Final R indexes [all data]	R1 = 0.0310, wR2 = 0.0808
Largest diff. peak/hole (e·Å^{-3})	0.7/-0.66
Flack parameter	0.368(17)

Table S2. Crystallographic parameters and refinement data for ScIrPF-13.

Crystallographic parameters	ScIrPF-13
Empirical formula	$C_{42}H_{18}Sc_{2.8}Ir_{2}N_{6}O_{28.8}$
Formula weight (g/mol)	1577.56
Crystal system	Orthorhombic
Space group	$Cmc2_1$
a (Å)	25.4624(5)
b (Å)	24.3673(5)
c (Å)	9.7884(2)
α (°)	90
β (°)	90
γ (°)	90
Volume (Å^3)	6073.2(2)
Z	4
Calculated density (g/cm^3)	1.725
Absorption coefficient (mm^{-1})	11.602
F(000)	3021.0
Radiation	CuKα (λ = 1.54178)
2θ range for data collection (°)	5.02 to 130.224
Index ranges	$-29 \leq h \leq 29$, $-28 \leq k \leq 26$, $-11 \leq l \leq 11$
Reflections collected	17162
Independent reflections	5003 [R_{int} = 0.0304, R_{sigma} = 0.0273]
Data/restraints/parameters	5003/97/388
Goodness-of-fit on F^2	1.087
Final R indexes [I>2σ (I)]	R_1 = 0.0327, wR_2 = 0.0761
Final R indexes [all data]	R_1 = 0.0369, wR_2 = 0.0784
Largest diff. peak/hole (e·Å^{-3})	1.29/-0.86
Flack parameter	0.47(2)

Table S3. Synthetic conditions tested during the synthetic screening for InIrPF-13. A= indium (III) acetate; N = indium (III) nitrate hexahydrate ; C = indium (III) chloride tetrahydrate; F = indium (III) fluoride; AC = indium (III) acetylacetonate; AcOH = acetic acid. Aging = the linker and the iridium salt were left stirring in water at room temperature for 24 hours.

Entry	mmol 2,5-PDC	mmol Indium salt	Indium salt	mmol $IrCl_3xnH_2O$	Temp. (ºC)	Time (h)	Solvent	mL	Modulator
1	0.24	0.12	A	0.08	170	24	H_2O	13	-
2	0.24	0.10	A	0.06	150	24	H_2O	13	-
3	0.20	0.21	A	0.13	150	24	H_2O	13	-
4	0.20	0.16	A	0.12	150	24	H_2O	13	-
5	0.20	0.16	A	0.10	150	24	H_2O	13	-
6	0.20	0.16	A	0.08	150	24	H_2O	13	-
7	0.20	0.16	A	0.06	150	24	H_2O	13	-
8	0.20	0.16	A	0.04	170	24	H_2O	13	-
9	0.20	0.16	A	0.04	170	24	DMSO	14	-
10	0.20	0.16	A	0.04	150	24	H_2O	13	-
11	0.20	0.12	A	0.04	150	24	H_2O	13	-
12	0.20	0.10	A	0.06	170	24	H_2O	13	-
13	0.20	0.10	A	0.06	150	144	H_2O	13	-
14	0.20	0.10	A	0.06	150	72	H_2O	13	-
15	0.20	0.10	A	0.06	150	24	H_2O	13	-
16	0.20	0.10	A	0.06	150	24	DMF	13	-
17	0.20	0.10	A	0.06	150	24	EtOH	13	-
18	0.20	0.10	N	0.06	150	24	H_2O	13	-
19	0.20	0.10	C	0.06	150	24	H_2O	13	-
20	0.20	0.10	A	0.06	150	24	H_2O	10	-
21	0.20	0.10	A	0.06	150	24	H_2O	8	-
22	0.20	0.10	A	0.06	150	24	H_2O	5	-
23	0.20	0.10	A	0.06	150	24	H_2O+EtOH	8+5	-
24	0.20	0.10	A	0.06	150	24	H_2O+EtOH	6.5+6.5	-
25	0.20	0.10	A	0.06	150	5	H_2O	13	-
26	0.20	0.10	A	0.06	150	3	H_2O	13	-
27	0.20	0.10	A	0.06	130	24	H_2O	13	-
28	0.20	0.10	A	0.04	150	24	H_2O	13	-
29	0.20	0.08	A	0.04	150	24	H_2O	13	-
30	0.20	0.06	A	0.04	150	24	H_2O	13	-
31	0.20	0.04	A	0.04	150	24	H_2O	13	-
32	0.20	0.02	A	0.04	150	24	H_2O	13	-
33	0.16	0.16	A	0.04	170	168	H_2O	13	-
34	0.16	0.16	A	0.04	170	72	H_2O	13	-
35	0.16	0.16	A	0.04	170	48	H_2O	13	-
36	0.16	0.16	A	0.04	170	24	H_2O	13	-
37	0.16	0.16	A	0.04	170	Aging+24	H_2O	13	-
38	0.16	0.16	A	0.04	150	24	H_2O	13	-

39	0.16	0.12	A	0.08	170	72	H_2O	13	-
40	0.16	0.12	A	0.04	150	24	H_2O	13	-
41	0.16	0.10	A	0.06	150	24	H_2O	13	-
42	0.16	0.08	A	0.12	170	72	H_2O	13	-
43	0.16	0.08	A	0.04	170	24	H_2O	13	-
44	0.16	0.08	A	0.04	150	24	H_2O	13	-
45	0.16	0.06	A	0.04	150	24	H_2O	13	-
46	0.16	0.04	A	0.08	170	24	H_2O	13	-
47	0.16	0.04	A	0.04	170	168	H_2O	13	-
48	0.16	0.04	A	0.04	170	48	H_2O	13	-
49	0.16	0.04	A	0.04	170	Aging+24	H_2O	13	-
50	0.16	0.04	A	0.04	170	24	H_2O	13	-
51	0.16	0.04	A	0.04	150	24	H_2O	13	-
52	0.12	0.16	A	0.04	150	24	H_2O	13	-
53	0.12	0.12	A	0.08	170	24	H_2O	13	-
54	0.12	0.12	A	0.04	150	24	H_2O	13	-
55	0.12	0.08	A	0.04	150	24	H_2O	13	-
56	0.12	0.06	A	0.04	150	24	H_2O	13	-
57	0.12	0.04	A	0.08	170	24	H_2O	13	-
58	0.12	0.04	A	0.04	150	24	H_2O	13	-
59	0.08	0.16	A	0.04	150	24	H_2O	13	-
60	0.08	0.12	A	0.04	150	24	H_2O	13	-
61	0.08	0.08	A	0.04	150	24	H_2O	13	-
62	0.08	0.06	A	0.04	150	24	H_2O	13	-
63	0.08	0.04	A	0.04	150	24	H_2O	13	-
64	0.06	0.16	A	0.04	150	24	H_2O	13	-
65	0.06	0.12	A	0.04	150	24	H_2O	13	-
66	0.06	0.08	A	0.04	150	24	H_2O	13	-
67	0.06	0.06	A	0.04	150	24	H_2O	13	-
68	0.06	0.04	A	0.04	150	24	H_2O	13	-
69	0.2	0.17	A	0.04	150	24	H_2O	13	0.05 mL Et_3N
70	0.2	0.10	A	0.06	150	24	H_2O	13	500 µL HNO_3 cc
71	0.2	0.10	A	0.06	150	24	H_2O	13	300 µL HNO_3 cc
72	0.2	0.10	A	0.06	150	24	H_2O	13	100 µL HNO_3 cc
73	0.2	0.10	A	0.06	150	24	H_2O	13	50 µL HNO_3 cc
74	0.2	0.10	A	0.06	150	24	H_2O	13	500 µL AcOH cc
75	0.2	0.10	A	0.06	150	24	H_2O	13	300 µL AcOH cc
76	0.2	0.10	A	0.06	150	24	H_2O	13	100 µL AcOH cc
77	0.2	0.10	A	0.06	150	24	H_2O	13	50 µL AcOH cc
78	0.2	0.10	A	0.06	150	24	H_2O	13	500 µL NaOH 1M
79	0.2	0.10	A	0.06	150	24	H_2O	13	300 µL NaOH 1M
80	0.2	0.10	A	0.06	150	24	H_2O	13	100 µL NaOH 1M
81	0.2	0.10	A	0.06	150	24	H_2O	13	50 µL NaOH 1M
82	0.2	0.06	A	0.06	150	24	H_2O	13	100 µL HNO_3 cc
83	0.2	0.06	A	0.06	150	24	H_2O	13	10 µL HNO_3 cc
84	0.2	0.06	A	0.06	150	24	H_2O	13	0.2 mmol Oxalic acid

85	0.2	0.04	A	0.04	190	24	H_2O	13	100 µL AcOH cc
86	0.2	0.04	A	0.04	180	24	H_2O	13	100 µL AcOH cc
87	0.2	0.04	A	0.04	170	24	H_2O	13	100 µL AcOH cc

Table S4. Synthetic conditions tested in the synthetic screening of InIrPF-13, using stock solutions of the organic linker as a starting point. Stock solutions were prepared in the following concentrations: 0.0042 M for H_2O; 0.0085 M for EtOH. A= indium (III) acetate; N = indium (III) nitrate hexahydrate ; C = indium (III) chloride tetrahydrate; F = indium (III) fluoride; AC = indium (III) acetylacetonate; AcOH = acetic acid; Aging = the linker and the iridium salt were left stirring in water at room temperature for 24 hours; the indium salt was added afterwards, and the solution was heated up to the specified temperature for each case.

Entry	Stock solution	mmol 2,5-PDC	mmol Indium salt	Indium salt	mmol $IrCl_3xnH_2O$	Temp. (ºC)	Time (h)	Modulator
1	12.5 mL EtOH	0.1	0.08	A	0.02	150	72	-
2	12.5 mL EtOH	0.1	0.08	A	0.02	150	24	-
3	12.5 mL H_2O	0.05	0.06	A	0.01	170	24	-
4	12.5 mL H_2O	0.05	0.05	A	0.01	150	24	-
5	12.5 mL H_2O	0.05	0.045	A	0.01	150	24	-
6	12.5 mL H_2O	0.05	0.04	A	0.02	170	24	-
7	12.5 mL H_2O	0.05	0.04	A	0.01	180	24	-
8	12.5 mL H_2O	0.05	0.04	A	0.01	180	Aging+24	-
9	12.5 mL H_2O	0.05	0.04	A	0.01	170	72	-
10	12.5 mL H_2O	0.05	0.04	A	0.01	170	Aging+72	-
11	12.5 mL H_2O	0.05	0.04	A	0.01	170	48	-
12	12.5 mL H_2O	0.05	0.04	A	0.01	170	24	-
13	12.5 mL H_2O	0.05	0.04	A	0.01	170	Aging+24	-
14	12.5 mL H_2O	0.05	0.04	A	0.01	150	72	-
15	12.5 mL H_2O	0.05	0.04	A	0.01	150	24	-
16	12.5 mL H_2O	0.05	0.04	N	0.01	150	24	-
17	12.5 mL H_2O	0.05	0.04	C	0.01	150	24	-
18	12.5 mL H_2O	0.05	0.04	F	0.01	150	24	-
19	12.5 mL H_2O	0.05	0.04	AC	0.01	150	24	-
20	12.5 mL H_2O	0.05	0.04	A	0.01	150	5	-
21	12.5 mL H_2O	0.05	0.04	A	0.01	150	3	-
22	12.5 mL H_2O	0.05	0.037	A	0.01	170	48	-
23	12.5 mL H_2O	0.05	0.035	A	0.01	150	24	-
24	12.5 mL H_2O	0.05	0.031	A	0.01	170	48	-
25	12.5 mL H_2O	0.05	0.03	A	0.01	150	24	-
26	12.5 mL H_2O	0.05	0.27	A	0.01	170	48	-
27	12.5 mL H_2O	0.05	0.25	A	0.01	150	24	-
28	12.5 mL H_2O	0.05	0.21	A	0.01	170	48	-
29	12.5 mL H_2O	0.05	0.20	A	0.01	190	24	-
30	12.5 mL H_2O	0.05	0.20	A	0.01	180	24	-
31	12.5 mL H_2O	0.05	0.20	A	0.01	170	72	-
32	12.5 mL H_2O	0.05	0.20	A	0.01	170	24	-
33	12.5 mL H_2O	0.05	0.16	A	0.01	170	48	-
34	12.5 mL H_2O	0.05	0.11	A	0.01	170	48	-
35	12.5 mL H_2O	0.05	0.01	A	0.01	170	48	-
36	12.5 mL H_2O	0.05	0.01	N	0.01	170	48	-
37	12.5 mL H_2O	0.05	0.01	C	0.01	170	48	-

38	12.5 mL H_2O	0.05	0.01	F	0.01	170	48	-
39	12.5 mL H_2O	0.05	0.01	AC	0.01	170	48	-
40	12.5 mL H_2O	0.05	0.05	A	0.01	170	24	100 µL AcOH cc
41	12.5 mL H_2O	0.05	0.04	A	0.01	170	24	50 µL AcOH cc
42	12.5 mL H_2O	0.05	0.04	A	0.01	150	24	300 µL AcOH 6M
43	12.5 mL H_2O	0.05	0.04	A	0.01	150	24	100 µL AcOH 6M
44	12.5 mL H_2O	0.05	0.04	A	0.01	150	24	50 µL AcOH 6M
45	12.5 mL H_2O	0.05	0.04	A	0.01	150	24	10 µL AcOH 6M
46	12.5 mL H_2O	0.05	0.02	A	0.01	170	24	300 µL HNO_3 cc
47	12.5 mL H_2O	0.05	0.02	A	0.01	170	24	100 µL HNO_3 cc
48	12.5 mL H_2O	0.05	0.02	A	0.01	170	24	50 µL HNO_3 cc
49	12.5 mL H_2O	0.05	0.02	A	0.01	170	24	300 µL AcOH cc
50	12.5 mL H_2O	0.05	0.02	A	0.01	170	24	100 µL AcOH cc
51	12.5 mL H_2O	0.05	0.02	A	0.01	170	24	50 µL AcOH cc

Table S5. Additional photocatalytic tests carried out with ScIrPF-13.

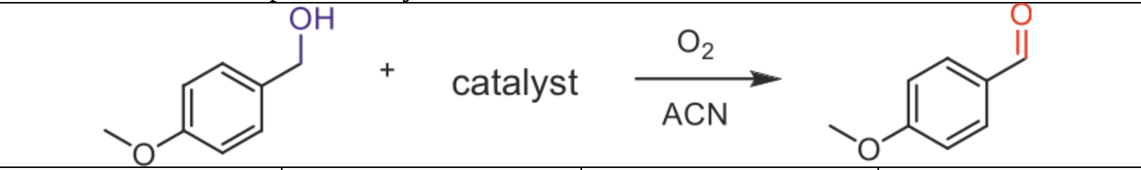

Time	mol % weight	% conversion	% selectivity
51h	2	43.9	100
30h	8	54.8	100
36h	8	56.0	100

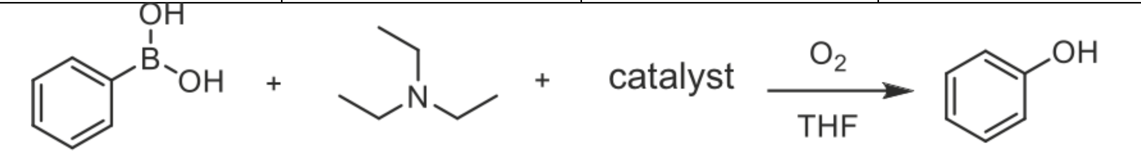

Time	mol % weight	Triethylamine	% conversion	% selectivity
48h	5	3 equiv.	54.6	100
24h	5	No	22.4	100

Table S6. FT-EXAFS fitting parameters for the fresh InIrPF-13 and ScIrPF-13 and recovered ScIrPF-13 samples. The fits were performed in the R-space over the (1.1–2.5) Å interval on the $k^{1,2,3}$-weighted $\chi(k)$ functions Fourier-transformed in the (4.0–12.0) $Å^{-1}$ *k*-space interval.

Sample/Condition	$R_{Ir\text{-}N\ (Ir\text{-}O)}$ (Å)	N	σ^2	ΔE	*R*-factor
Fresh InIrPF-13	2.05 ± 0.05	7.00 ± 1.85	0.002 ± 0.002	12.5 ± 3.7	0.049
Fresh InIrPF-13 under light	2.03 ± 0.03	6.98 ± 1.01	0.002 ± 0.001	10.1 ± 2.1	0.014
Fresh InIrPF-13 after light	2.02 ± 0.02	7.32 ± 0.96	0.002 ± 0.001	8.1 ± 1.9	0.012
Fresh ScIrPF-13	1.98 ± 0.03	7.28 ± 1.24	0.003 ± 0.001	6.1 ± 6.0	0.096
Fresh ScIrPF-13 under light	2.03 ± 0.03	6.68 ± 1.42	0.002 ± 0.002	9.3 ± 3.1	0.036
Fresh ScIrPF-13 after light	2.05 ± 0.10	7.81 ± 1.68	0.003 ± 0.002	11.6 ± 3.0	0.031
Recovered ScIrPF-13	2.02 ± 0.08	6.29 ± 1.01	0.003 ± 0.001	7.5 ± 1.6	0.008
Recovered ScIrPF-13 under light	2.03 ± 0.09	6.47 ± 1.33	0.003 ± 0.001	9.5 ± 2.2	0.014
Recovered ScIrPF-13 after light	2.04 ± 0.09	6.56 ± 1.42	0.003 ± 0.002	9.65 ± 2.34	0.017

References.

(1) Guo, Z.; Li, Y.; Yuan, W.; Zhu, X.; Li, X.; Cao, R. Syntheses, Structures, and Characterizations of Two New Indium(III) Compounds from 1D ⋯In-OH-In-OH⋯ Chains and Pyridinedicarboxylic Ligands. *Eur J Inorg Chem* **2008**, No. 8, 1326–1331. https://doi.org/10.1002/ejic.200701060.

(2) Ravel, B.; Newville, M. ATHENA, ARTEMIS, HEPHAESTUS: Data Analysis for X-Ray Absorption Spectroscopy Using IFEFFIT. *J Synchrotron Radiat* **2005**, *12* (4), 537–541. https://doi.org/10.1107/S0909049505012719.

(3) Camp, C. H. PyMCR: A Python Library for Multivariatecurve Resolution Analysis with Alternating Regression (MCR-AR). *J Res Natl Inst Stand Technol* **2019**, *124*. https://doi.org/10.6028/jres.124.018.

(4) Newville, M. Larch: An Analysis Package for XAFS and Related Spectroscopies. *J Phys Conf Ser* **2013**, *430* (1), 12007. https://doi.org/10.1088/1742-6596/430/1/012007.
